# Tissue-associated microbial detection in cancer using human sequencing data

**DOI:** 10.1186/s12859-020-03831-9

**Published:** 2020-12-03

**Authors:** Rebecca M. Rodriguez, Vedbar S. Khadka, Mark Menor, Brenda Y. Hernandez, Youping Deng

**Affiliations:** 1 Bioinformatics Core, Department of Quantitative Health Sciences, John A. Burns School of Medicine, University of Hawaii, Mānoa, Honolulu, HI USA; 2 Epidemiology, University of Hawaii Cancer Center, University of Hawaii, Honolulu, HI USA; 3 Population Sciences in the Pacific Program-Cancer Epidemiology, Honolulu, HI USA; 4 NIDDK Central Repository, National Institute of Diabetes and Digestive and Kidney Diseases, NIH, Bethesda, USA

**Keywords:** Cancer microbiome, Computational frameworks, NGS

## Abstract

Cancer is one of the leading causes of morbidity and mortality in the globe. Microbiological infections account for up to 20% of the total global cancer burden. The human microbiota within each organ system is distinct, and their compositional variation and interactions with the human host have been known to attribute detrimental and beneficial effects on tumor progression. With the advent of next generation sequencing (NGS) technologies, data generated from NGS is being used for pathogen detection in cancer. Numerous bioinformatics computational frameworks have been developed to study viral information from host-sequencing data and can be adapted to bacterial studies. This review highlights existing popular computational frameworks that utilize NGS data as input to decipher microbial composition, which output can predict functional compositional differences with clinically relevant applicability in the development of treatment and prevention strategies.

## Introduction

Cancer is one of the leading causes of morbidity and mortality in the globe. Annually an estimated 14.1 million are diagnosed, and 8.2 million die from cancers around the world. In the United States alone, 1.7 million cases are diagnosed, and about six hundred thousand die from the disease [1–3]. Cancer is a multifactorial disease with known genetic and environmental etiologies. Microbiological infections account for up to 20% of the total global cancer burden [4, 5]. Viruses are commonly attributed and are responsible for at least 10% of all human cancers [6]. Multiple studies have evaluated viral content and its influence on cancer pathogenesis utilizing advanced technologies and bioinformatics approaches.

Meanwhile, recent limited evidence exists proposing relationships between bacterial species and disease either as effector or consequence of tumorigenesis. While much effort has gone into characterizing cavity organs microbiota, that of solid tumors is less explored. The characterization of tissue-associated microbiota is challenging as well as computationally intensive. Next-generation sequencing technologies provide an opportunity to explore better bioinformatics approaches to detect microbial agents and can assist in the interpretation of not only viral but bacterial species impact in tumor tissue. The examination of microbial species is pivotal to developing new prevention and treatment strategies.

## Relationship of microbiota with cancer pathogenesis

The human microbiome, defined as the aggregation of microorganisms that live in and on our bodies, contributes to our broader genetic portrait [7, 8]. The microbiota within each organ system is distinct, which can drive functionally relevant inter-individual variations and determinants of disease [7, 9–12]. Microbial community variations, production of bacterial metabolites, and microbial interactions with the human host have been attributed to detrimental and beneficial tumoral effects since the eighteenth century [13, 14]. This highlights the unique agonistic and antagonistic effects of the human microbiome in cancer progression and has become an area of intense exploration. While contribution by some viral pathogens is firmly established, the role of the bacterial community remains controversial. The mechanisms by which viral agents contribute to pathogenesis have been reviewed in detail and are not covered here [15–18]. Mechanisms by which bacteria contribute to the alterations and the carcinogenic process are not all well understood. It is known, however, that similar to viruses, persistent and chronic infections may initiate the process or promote established cancers [14, 19–22]. Alteration of the bacterial community could also result in beneficial effects on the tumor microenvironment. In fact, according to the literature, any agent capable of stimulating host immune defenses can minimize the incidence and be advantageous to established tumors. Modification of the immune cascade in response to infection or dysbiosis is one of the most critical aspects of tumor-microenvironment cross-talk [23, 24]. Altered host-dynamics can increase bacterial translocation as a direct consequence of changes in microbial composition, resulting in increased inflammation. Bacterial products and bacterial metabolites may have protective effects on survival, reduced growth of cancer cells, or modulate anticancer immunosurveillance at local or distant sites [10]. Butyrate for example, which has anti-inflammatory properties, is thought to be protective while secondary bile acids are considered carcinogenic [25, 26]. These variations in the microbial composition may be directly or indirectly responsible for the carcinogenic process in susceptible populations, alter the course of established cancer, or influence therapeutic response and can assist in understanding patient inter-variability [27, 28].

New microbial (viral, bacterial, and other pathogens) contributions to cancer, whether beneficial or detrimental, are being discovered. Improved techniques and integrated data networks facilitate discoveries and have become the focus of multiple studies [29–37]. Recent studies have found that specific bacterial taxa are consistently identified in tumor tissue [38]. Compared to adjacent or control tissue, *Fusobacteria*, *Alistipes*, *Porphyromonadaceae*, *Coriobacteridae*, *Staphylococcaceae*, *Akkermansia*, and *Methanobacteriales* are found at increased levels in tumor, while *Bifidobacterium*, *Lactobacillus*, *Ruminococcus*, *Faecalibacterium*, *Roseburia*, and *Treponema* are at decreased levels [38–46] (Table 1). Also, viral and bacterial co-occurrence is thought to modulate tumor aggressiveness [47–49]. Based on epidemiological and geographic correlations analyses, it is suggested that viral agents interact with bacteria resulting in more aggressive tumors. For example, stomach tumors infected with Epstein Barr virus are recognized to be molecularly distinct. Meanwhile, Epstein Barr virus is thought to interact with *Helicobacter pylori* driving aggressiveness*,* however insufficient evidence exists. In hepatocellular carcinoma viral co-infection with HBV or HCV and the interaction between the proteins, HBx HCV core and NS5a, can also lead to more aggressive tumors. Interaction with other exposures, alcohol consumption, smoking, co-morbidities, betel nut chewing can act as co-factors altering the tumor microenvironment in cancers of the head and neck [50].

**Table 1:** Known and suspected microbial association with cancer pathogenesis

Cancer type	Known microbial associations	Suspected agents	References
Breast Triple-negative, HER2+, ER+	None	Epstein–Barr virus, human papillomaviruses *Alistipes* spp. *Bacteroides fragilis*, *Sphingobium yanoikuyae*, *Microbial dysbiosis*	[35, 36, 39, 40]
Prostate Prostate adenocarcinoma	None	*Cutibacterium acnes* *Bacteroides massiliensis* *Streptococcus* spp. *Staphylococcus* spp. Microbial dysbiosis	[37, 41, 42]
Stomach Stomach adenocarcinoma	*Helicobacter pylori,* Epstein Barr Virus	Microbial dysbiosis	[57, 70]
Liver Liver and intrahepatic bile duct	Hepatitis viruses, Parasitic infections	*Helicobacter pylori*	[43]
Cervical Cervical squamous cell and endometrial carcinoma	Human papillomaviruses	*Chlamydia trachomatis*, microbiome dysbiosis	[63]
Head and Neck Oropharyngeal and laryngeal	Epstein Barr Virus, Human papillomaviruses	*Fusobacterium nucleatum*, microbiome dysbiosis	[56, 58]
Colon and rectum Colorectal adenocarcinoma	Microbial dysbiosis *Fusobacterium nucleatum*	Human papillomavirus *Helicobacter pylori*, *Streptococcus bovis*, E. *Escherichia coli*, *E. Bacteroides fragilis*, *Campylobacter* spp.	[10, 31, 32, 55]
Kidney Renal cell carcinoma and clear cell carcinoma	None	Hepatitis C virus Epstein Barr Virus Urinary tract infection-associated pathogens	[44]
Lung Lung squamous cell and adenocarcinomas	None	Epstein Barr Virus Molluscum Contagiosum virus Microbial dysbiosis *Chlamydia pneumoniae*	[45]
Bladder Bladder squamous cell carcinoma	*Schistosoma haematobium*	Human papillomavirus Epstein–Barr Virus	[46]

Common cancer types listing known and suspected microbial (viral, bacterial, and other) agents associated with cancer pathogenesis or that have been identified as common causes of infection in cancer patients, which may play a role in patient inter-variability

Competitive interaction between viral-bacterial species and other exposures may be more apparent at broader taxonomic levels. Taxonomic level analyses of the gut, oral, and other cavity organ microbiomes reveal bacterial candidates associated with pathology of disease [33, 35, 51]. These findings could be applied to preventive or complementary therapies. Questions remain, whether microbial composition findings derived from surrogate material, like stool and saliva within these cavity organs, directly relate to the microbial composition within the solid tumor tissue and surrounding tumor microenvironment. Further, whether the tissue-associated tumor microbial composition can be consistently derived from existing human sequencing data and how to best discern microbial roles in inter-population variability. Identification of microbial composition directly from tumor tissue human sequences enables not only the study of microbial changes and cancer pathogenesis but microbial genomic integration [34]. Integration of microbial DNA into the human genome may prove key in the identification of passager versus driver bacteria in cancer pathogenesis.

## Microbiome detection in high throughput sequencing data

Next-generation sequencing (NGS) technologies, also known as high-throughput, provide a powerful tool for the evaluation of the role of microbes in cancer development and progression as well as differences across populations. NGS is a useful and unbiased tool that can be used for the identification of previously undetected or unsuspected causative microorganisms in molecular diagnostics [52]. It has become vital and necessary for the integrative analysis of cancer biology, enabling description of the mutational and molecular landscape of cancer for both direct and indirect taxonomic studies [53]. These techniques take advantage of NGS production of short reads and the predominance of host-derived sequences to examine pathogen-host interaction, including their correlation with metabolic and regulatory mechanisms in cancer [30, 32, 54–58]. Although the establishment of a causal relationship requires a more detailed characterization of the tumor microbiota and microbial population dynamics, integration of host sequencing data with clinical and epidemiological data can provide valuable information to the understanding of the role bacteria play in cancer pathogenesis and population differences. Given the close interaction between microbes and the host responses, it is essential to identify the compositional structure and clinically relevant functional pathways with an integrated approach.

## Computational frameworks and tissue-associated bacteria detection in cancer

**Fig. 1:**
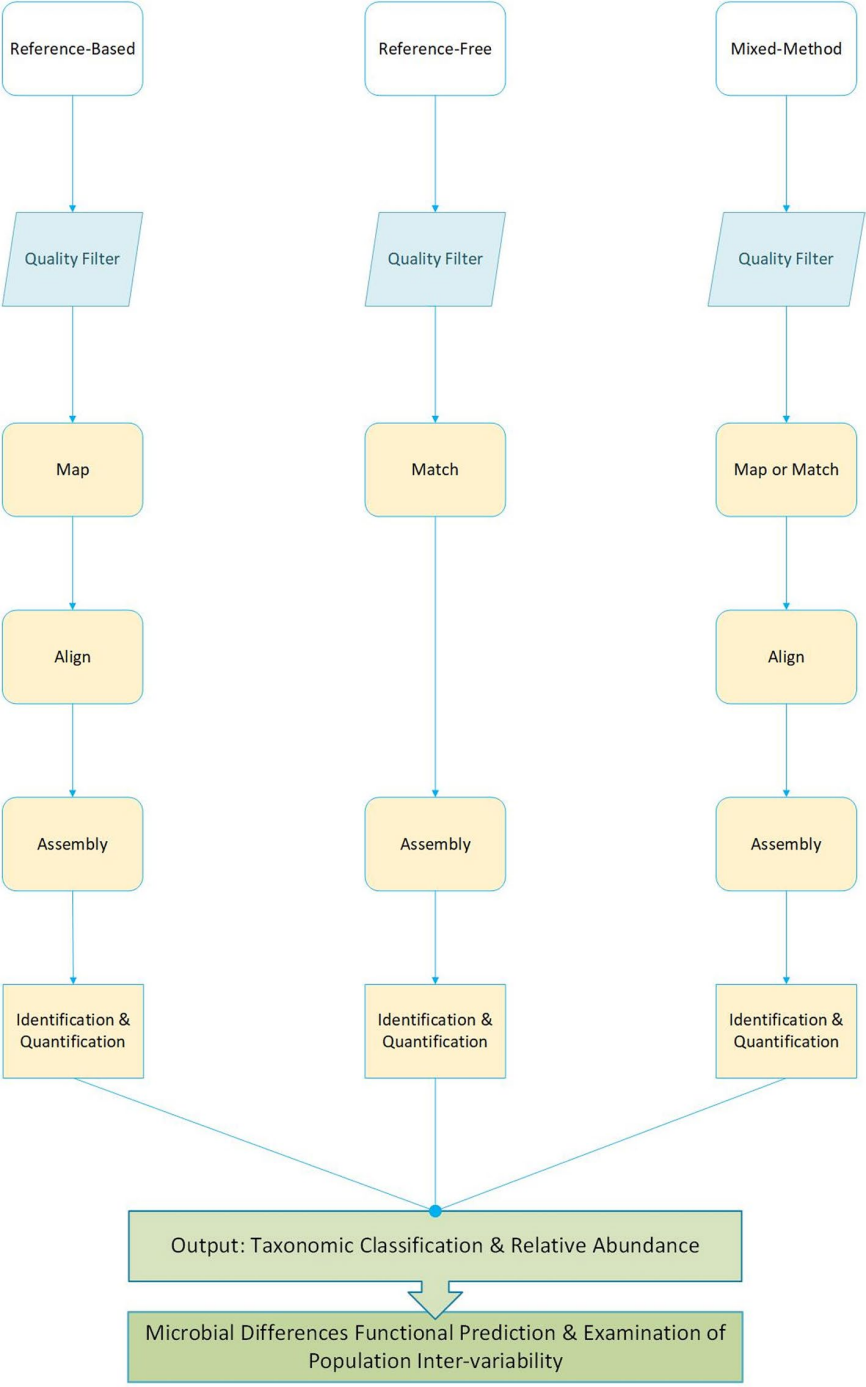
Generic pipeline comparing three basic computational frameworks designed to identify microbial reads from human sequences. Generic pipelines can be summarized into three general stages, pre-processing (blue), processing (yellow), and analyses post-processing (green). During pre-process, most methodologies trim and quality filter sequencing reads. Quality reads are mapped and aligned during the processing steps to either human or pathogen reference sequences or key identifying factors before making a final identification call. Once species have been identified, their composition is characterized in detail, depending on the methodology being used. Finally, having taxonomic classification and compositional structure permits downstream correlation analyses and functional-relevant identification of molecular pathways. Differential functional prediction and patient inter-variability aid in the identification of novel microbe based prevention and treatment strategies

Bioinformatics computational frameworks are methods and pipelines able to accommodate user-defined parameters and deliverables to understand the basis of biological concepts [59]. Mining NGS data using bioinformatics computational frameworks provide great opportunities in understanding the role of bacteria in cancer pathogenesis. Numerous state-of-the-art bioinformatics tools and methods are available today that support the identification of microbial novel targets in cancer diagnostics, treatment, prevention, and control. Several studies have demonstrated that pathogenic and commensal bacteria composition can be derived from human tumor tissue utilizing various bioinformatics computational approaches by sequential filtering and matching steps [52, 60–63]. Pathogen detection derived from human sequences has been primarily completed by computational subtraction with one of three approaches, reference-based, reference-free, or mixed methods with one primary core pipeline involving the removal of human-host sequences to characterize remaining sequencing reads (Fig. 1). Pathogen detection algorithms may be classified by (1) their methodology, (2) the order in which human sequencing reads are identified and removed, and (3) what happens with the remaining sequences (whether these go through *de-novo* assembly or are filtered out). Here, we discuss ten computational frameworks, PathSeq, SRSA, CaPSID, PathoScope 2.0, SURPI, VirusScan, MetaShot, ConStrains, RINS, and GRAMMY, designed to identify microbiota (virus, bacteria, and other) derived from human sequences with applications in human cancer (Table 2). Computational frameworks that strictly match sequencing reads to pathogen libraries or those designed for direct metagenomics analyses are not included (see Nooij et al. 2018 for a recent in-depth review of these tools [64]).

**Table 2:** Computational frameworks designed to detect microbiota from human sequences by subtractive, filtration, or mixed methods

Framework	Approach	Dependencies	Input | output	Advantages/disadvantages	Cancer validation	Refs.
PathSeq	Alignment and de novo assembly	BLAST BLASTN BLASTX MAQ MegaBLAST RepeatMasker Velvet	Input: RNA-seq or DNA-seq Output: Pathogen presence/absence	Scalable cloud computing Feasible for known and novel pathogen identification Two-pass subtraction with increased filtering costs	Cervical cancer (cell line and simulated data) TCGA ovarian	[63, 68]
SRSA	Alignment and de novo assembly	Velvet MegaBLAST BLAST BWA TopHat	Input: RNA-seq Output: Species-level taxonomy characterization (prevalence)	Incorporates sample pre-processing, quality filtering, sequence mapping, and assembly Not freely available No known updates Original work validation was limited to cell line	HIV-1 cell line	[60]
CaPSID	Mix-method, simultaneous alignment, filtration and de novo assembly	BioPython Bowtie2 Trinity	Input: RNA-seq or DNA-seq Output: Top-hit pathogen genome identification ranked by maximum gene coverage	Web-based, open-source and scalable application; Modular analyses; Single pass filtering, which may fail to subtract host reads	Ovarian cancer TCGA stomach	[67]
SURPI	Dual scanning mode; Known pathogens identification or de novo assembly	SNAP RAPSearch BWA BLASTN Bowtie2 DUST in PRINSEQ	Input: Paired-end metagenomic Output: Species-level taxonomic classification and coverage map	Scalable to cloud or standalone servers Capacity to incorporate reference database Dual-mode: quantitative and semi-quantitative pathogen identification	Prostate cancer (cell line, tissue biopsies) Colorectal cancer (tissue biopsies)	[71]
PathoScope 2.0	Penalized probabilistic identification; Modular filtration, alignment and assignment	SAMtools BLASTX Bowtie2 thetaPrior	Input: Metagenomic or genomic (RNA-seq or DNA-seq) Output: Strain level pathogen relative abundance	Modular detailed result reporting with Designed for low abundance strain-level identification MySQL server required; no connection to the population structure of relevant species	TCGA stomach	[69, 70]
VirusScan	Identification of known viral and integration sites	BWA BLAST MegaBLAST Pindel RepeatMasker PHYLIP	Input: RNA-seq Output: Viral read abundance and integration sites	Designed for viral identification; Abundance and integration sites analyses	TCGA cancer cohorts	[72]
MetaShot	Two-step similarity filtering and taxonomic assessment	Bowtie2 TANGO STAR Bash	Input: RNA-Seq or DNA-Seq Output: Assigned read report and Krona plot with relative abundance	Extracts unassigned reads; Allow for functional annotations; Slower than other applications	None	[73]
ConStrains	Marker-based (SNP patterns) Strain-level prediction	MetaPhlAn PhyloPhlAn Bowtie2 SAMtools Metropolis-Hasting Monte-Carlo	Input: Metagenomics (RNA-seq) Output: Strain-level prediction and relative abundance	Single reference strain collection; Facilitates functional analyses when combined with reference genome-based gene coverage metadata	None	[74]
RINS	Intersection based identification and removal	Bowtie BLAST BLAT Trinity	Input: Mate-paired RNA-seq unmapped reads Output: Pathogen contigs	Requires prior knowledge of reference; Detection limited to user-defined parameters	Prostate cancer (cell line)	[66]
GRAMMy	Mix- model Bayesian, Expectation–Maximization and maximum likelihood estimation	BLAST BLAT MAQ Bowtie PerM BLASY	Input: Metagenomics reads Output: Genomic relative abundance as numerical vectors	User flexibility Probabilistic handling of ambiguous hits Computational efficiency	None	[76]

Comparison of computational workflows designed to derive microbial content from human sequences by subtractive and filtering methods, broadly categorized as reference-based, reference-free, and mixed methods approaches. Data requirements to run the pipeline, output information, as well as advantages and disadvantages for each, are summarized. Most have been validated with large cancer datasets, including TCGA sequencing data. ConStrains is based on reference-free, while all other approaches are reference-based or mixed-methods

In NGS, about 10% of the sequencing reads are flagged unmapped to the human genome after alignment [65]. Under the assumption that the sequenced tissue contains both host and microbial information, the bacterial composition can then be detected after the computational subtraction of human content [61–63]. Computational subtraction methods for microbial identification and discovery derived from human tissue were first introduced by Weber et al. and Xu et al. [61, 62]. These early approaches were computationally intensive and involved creation of a cDNA library with subsequent subtraction of human-expressed sequence tags [61, 62]. Newer methods take advantage of NGS data repositories’ unmapped-to-human sequences and have lower computational requirements. Frameworks that consider unmapped-to-human sequencing reads as input data can lower computational costs while facilitating novel discoveries.

Most computational subtraction frameworks are reference-based approaches [60, 63, 66, 67]. Reference-based, by definition, requires mapping to a reference, in this case, human host genome, then allocating all leftover unmapped-to-human reads to pathogen target genomes. PathSeq, for example, combines alignment and de novo assembly with a two-pass subtraction process [63]. It aligns the sequencing reads to target genomes and quantify their abundance based on the total number of aligned sequencing reads and the genome coverage, enabling identification of both commensals and pathogens whether known or novel. However, the two-pass filtration process may eliminate a high number of sequences, which may increase filtration costs and limit identification. PathSeq has been utilized in pathogen identification for various infection-associated and inflammation-associated cancers, notably the emerging association of *Fusobacterium nucleatum* in colorectal cancer [68]. SRSA, short RNA subtraction, and assembly utilize short RNA mapping and assembly to identify pathogens in relation to host-sequencing reads [60]. SRSA has the capability for use in microbial identification in infection-associated cancers. However, initial work was limited to mycoplasma detection in HIV-1 cell lines, and its computational methods are also not freely available. Unlike SRSA, CaPSID (computational pathogen sequence identification) is a web-based open-source platform that similar to PathSeq, performs mapping and de novo assembly [67]. CaPSID differs in its single-pass alignment and filtration process, where both human and pathogen reads are aligned to reference genomes while separating those that do not match either for de novo assembly simultaneously. Its potential in cancer was demonstrated by Borozan et al. in stomach adenocarcinoma samples from TCGA and other cancer networks [49]. Borozan et al. evaluated human herpesvirus 4 (HHV-4) variants to determine oncogenic potential differences among samples from different country origins providing evidence of the potential of such frameworks in future population studies [49]. Unlike PathSeq, SRSA, and CaPSID, PathoScope 2.0 does not perform de novo assembly; instead, it utilizes penalized statistical mix-model and probabilistic pathogen identification [69]. It also provides detailed reports with core and optional module format that enable user customization. On the downside, the target reference genome must be present for precise identification of microbes. PathoScope 2.0 is designed to identify low abundant strains, making it an ideal tool for host-derived microbial analyses due to the low abundance of microbial reads in relation to host reads found in sequencing data. Zhang et al. incorporated PathoScope 2.0 methods with its WGS PathSeq-based methods for microbial relative abundance estimation of gastric cancer clinical samples and existing sequencing data [70]. SURPI, sequence-based ultra-rapid pathogen identification, was also designed for pathogen detection from clinical samples for surveillance similar to PathoScope 2.0. One of the advantages of SURPI is the capacity for quantitative and semi-quantitative simultaneous identification, meaning it can perform mapping and de novo assembly for divergent microbial analyses [71]. SURPI has been validated against samples from colon and prostate cancer-derived datasets. Unlike those before mentioned that were designed to identify various microorganisms, VirusScan is a referenced-based computational subtraction approach designed to profile the viral composition. It also calculates abundance and integration sites within human tumors utilizing unmapped-to-humans and poorly mapped to human genome reads [72]. This approach was used to identify population viral differences in TCGA’s liver and stomach cancer cohorts [72]. The inclusion of bacterial libraries could assist in future co-occurrence and tumor microbiome analyses. MetaShot is similar to prior mentioned reference-based approaches in that it shares a two-step filtration method to identify candidate pathogens; however, it is a bit more stringent in its taxonomic assignment [73]. This feature enables functional annotation with great potential in tissue-associated bacterial composition analyses. On the other hand, its rigorous approach comes with higher computational costs and has yet to be validated in cancer datasets.

Other methods may utilize pre-defined target genomic markers like k-mers, single nucleotide polymorphisms (SNP), or unique sequence tag libraries to identify and retain pathogen information while removing human host sequences from further consideration. These approaches can be described as marker-based methods and are mostly considered reference-free. Reference-free, marker-based approaches such as ConStrains, conspecific strains rely on the creation of SNP profiles to predict pathogen strains contained within the sequencing sample [74]. However, methods such as this are not wholly reference-free, rather minimally reference-dependent [74]. ConStrains works by inferring microbial abundance of conspecific strains utilizing SNP patterns and de novo assembly with microbial prediction estimation based on Metropolis-Hasting Markov Chain Monte-Carlo model. Although ConStrains has not been used in cancer genomic data, it has the capability for functional analyses, which are pivotal in understanding different microbial effects in cancer pathogenesis, particularly those of infectious etiology.

Computational frameworks may also take advantage of mixed approaches which can be reference-free or reference-based. Reference-Free Mixed or mixture-model approach utilizes intersection analyses, while mixture-model approaches take advantage of both reference and marker-based methods. RINS, rapid identification of non-human sequences, uses intersection analysis. Similar to ConStrain is not completely reference-free. It employs a pre-defined query reference that includes genomes of viruses, bacteria, or other pathogens to find the intersect, rather than mapping and subtracting the human reference genome [66]. RINS has been validated in prostate cancer and has low computational requirements. However, it can only detect pathogens that are explicitly defined within the query reference [66]. By only being able to identify defined references expressly, it risks the removal of unknown sequences, hindering novel pathogen discovery. Mixture-model approaches differ from traditional computational subtraction in that these either maps against a pre-determined pathogen reference in series [66, 73], against both human and pathogen in parallel [75], or some combination of these before filtering out human-host sequences. Mixture-model approaches like GRAMMy, genome relative abundance estimation framework using mixture model theory, utilize expectation–maximization algorithms to calculate microbial genome relative abundance at different taxonomic levels [76]. GRAMMy is designed to use either mapping or de novo assembly in the absence of a reference genome [76].

## Computational pipelines and functional prediction of microbial differences

Recent works in the gut microbiome revealed the utility of taxonomic differences, epigenetic, heritable, and co-occurrence patterns in the understanding of cancer pathogenesis [77]. Microbial compositional differences and population variations have been thoroughly reviewed in [78]. From these and other works, we understand that accurate interpretation of microbial impact cancer pathogenesis involves more than compositional differences. Functional annotation and prediction of molecular processes are equally important in the identification of clinically relevant microbial interactions within the human host.

Post-processing pipelines have been developed to translate microbial composition outputs into predicted mechanisms through which bacteria may influence host immune responses, gene, and protein expression within the tumor microenvironment. For example, pipelines such as PICRUSt [79], Tax4Fun [80], and ShortBRED [81] can assist in the identification of functional annotations and subtle differences across populations within and across tumor types. Although these pipelines are designed to predict functional profiles derived from 16S rRNA sequencing data, they have application in host-derived microbial profiles when used in integrated approaches. For example, PICRUSt (Phylogenetic Investigation of Communities by Reconstruction of Unobserved States) infers microbial community host-associated functional composition based on gene annotation databases such as the Kyoto Encyclopaedia of Genes and Genomes (KEGG) or the Clusters of Orthologous Group (COGs) [82]. Tax4Fun (Taxonomy functional community profiling) on the other hand, predicts the functional capabilities of microbial communities based on 16S rRNA datasets. Tax4Fun provides an excellent approximation to functional profiles obtained from metagenomic shotgun sequencing approaches and has been successfully used to identify signs of ethnic acculturation in oral microbiota [80]. Both methods, in combination with computational frameworks designed to determine the microbial composition, provide insight into tumor-microbial associations and enable the discovery of new associations, the identification of patterns of co-occurrence, and possible host interaction effects. Gene and protein expression within the tumor and surrounding tissue information in conjunction with microbial composition may provide much-needed information on differential analyses. ShortBRED (Short, Better REad Dataset) is one that quantifies the abundance of functional gene families to predict protein profiles within the sample [81]. It can predict antibiotic resistance genes and virulence factors protein families that are pivotal in understanding therapeutic response. A combination of microbial detection and functional prediction approaches is critical, especially given the potential use in microbe-based prevention strategies and targeted therapies.

## Conclusions

There is a great diversity present in the human tumor microenvironment that makes identification of the microbial community challenging. Next generation sequencing technologies and the use of these computational tools permit the discovery of new microbes that are non-culturable and would otherwise remain undiscovered [83]. Profiling and characterization of the bacterial community and functional annotations can provide information on the effects of microbiota on colonized tissue, the progression of inflammation, alteration of cellular processes, and impact on tumor-promoting genes within the tumor microenvironment. Computational frameworks for microbial detection evaluated here are broadly classified as reference-based or reference-free, or mixed methods and mainly utilize computational subtraction that has been used or have the potential for such microbial diversity evaluations. These methodologies could help shed light on the role of the microbiota in cancer pathogenesis. Further, the output from these workflows combined with phylogenetic and protein-functional predictions from bioinformatics pipelines such as PICRUSt, Tax4Fun, and ShortBRED, among others, provide important clues in the understanding of microbial differences and commonalities and the potential impact on differential outcomes, therapeutic response, and population inter-variability. Recent works [84–86] demonstrate the utility of tissue-associated microbial detection derived from existing human sequencing data and the computational tools to characterize them. Differences may highlight effectors that impact the treatment decision making process and potential for targeted therapies. Their use should be promoted as first approach to the identification or confirmation of known, suspected, and novel pathogen associations in cancer.

## Data Availability

Not applicable.

## Abbreviations

CaPSID: Computational Pathogen Sequence Identification workflow
cDNA: Complementary DNA
COG: Cluster of Orthologous Group
ConStrains: Conspecific strain workflow
GRAMMy: Genome Relative Abundance Estimation framework
HBV: Human hepatitis virus B
HBx: Hepatitis viral X protein
HCV: Human hepatitis virus C
HCV core: Hepatitis viral core protein
HHV-4: Human herpes virus 4
HIV-1: Human immunodeficiency virus 1
KEGG: Kyoto Encyclopaedia of Genes and Genomes
MetaShot: Metagenomics Taxon Classification workflow
NGS: Next generation sequencing
NS5a: Non-structural protein 5A
PathoScope: Pathogen Identification and Quantitation modular workflow
PathSeq: Pathogen sequence workflow
PICRUSt: Phylogenetic Investigation of Communities by Reconstruction of Unobserved States
RINS: Rapid Identification of Non-human Sequence workflow
ShortBRED: Short Better Read Dataset
SNP: Single nucleotide polymorphism
SRSA: Short-RNA subtraction and assembly workflow
SURPI: Sequence Based Ultra Rapid Pathogen Identification workflow
Tax4Fun: Taxonomy functional community profiling
TCGA: The Cancer Genome Atlas
VirusScan: Viral sequence scanner workflow
WGS: Whole genome sequencing
